# Fast-Track Hemostasis by an Automated Fastener for Decannulation of an Antegrade Cardioplegia Cannula in Robotic Cardiac Surgery

**DOI:** 10.1016/j.atssr.2025.03.014

**Published:** 2025-04-02

**Authors:** Toshikuni Yamamoto, Tomonari Uemura, Yasunari Hayashi, Masato Mutsuga

**Affiliations:** 1 Department of Cardiac Surgery, Nagoya University Graduate School of Medicine, Nagoya, Aichi, Japan

## Abstract

In robotic cardiac surgery, many knot ties must be made with a knot pusher by the patient’s side surgeon. However, knot-pusher ties are characterized by weak attachment pressure. Therefore, we decided to use the Cor-knot automated fastener (LSI Solutions) to achieve hemostasis at the antegrade cardioplegia cannulation site. This technique allowed us to obtain speedy and reliable hemostasis at the antegrade cardioplegia cannulation site in robotic cardiac surgery.

The skill of a patient’s side surgeon is extremely important in robotic cardiac surgery. In particular, many knot ties must be made with a knot pusher. However, knot-pusher ties are characterized by weak attachment pressure.^1^ In robotic mitral valve surgery, a particular challenge is achieving hemostasis at the antegrade cardioplegia (ACP) cannulation site. Some techniques to obtain hemostasis at the ACP site have been reported.^2^ Use of the Cor-knot automated fastener (LSI Solutions) for hemostasis is a simple and fast-track technique to manage bleeding. Our technique enables hemostasis to be achieved independent of the patient’s side surgeon’s skill or the use of a knot pusher.

## Technique

**Figure 1:**
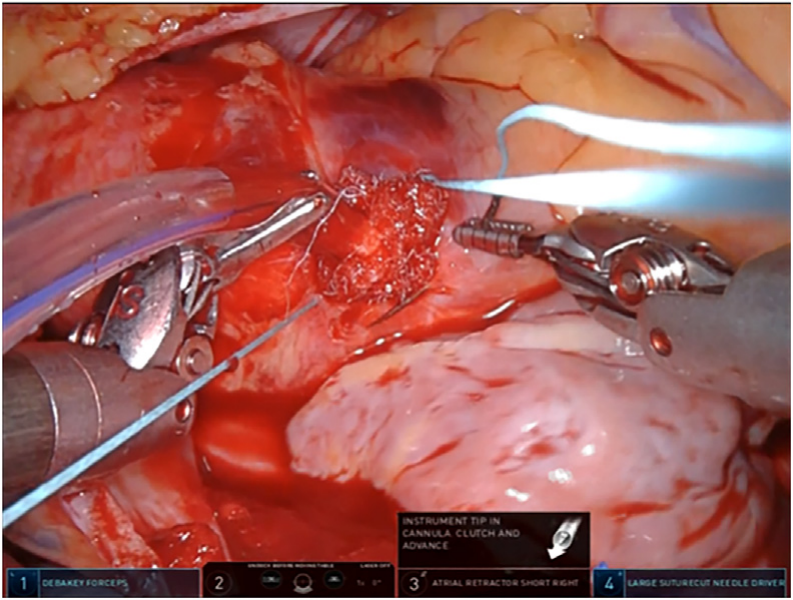
The pursestring suture is placed with a pledgeted 2-0 polyester suture that surrounds the antegrade cardioplegia site, with the needle running only to the adventitia.

**Figure 2:**
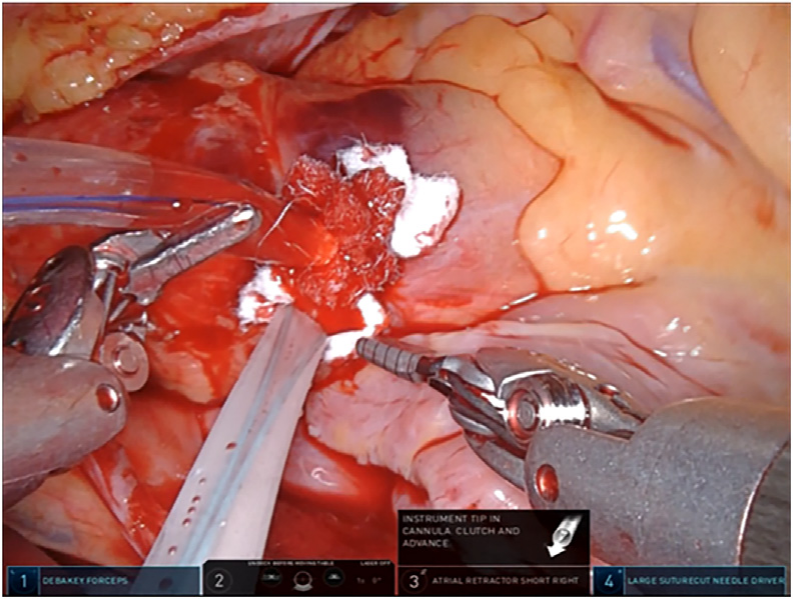
A tourniquet is used to tighten the outer thread after loosening the inner thread.

**Figure 3:**
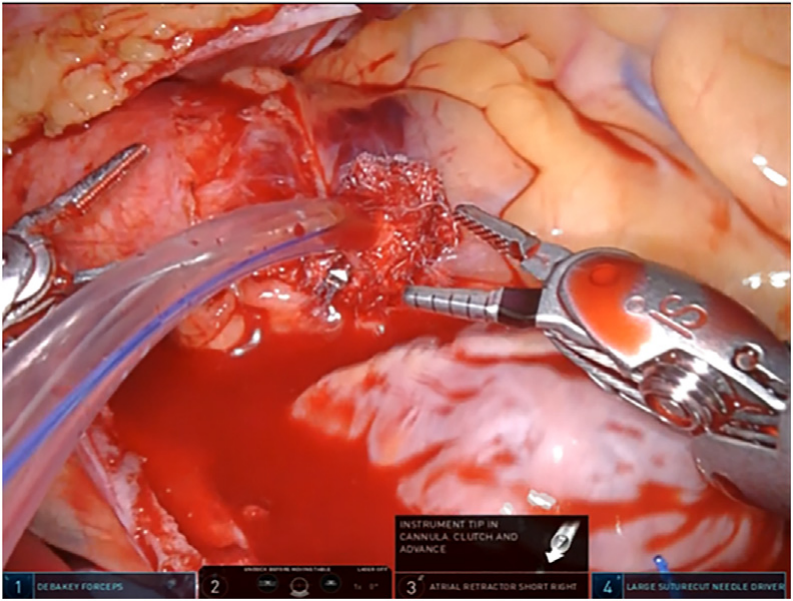
The Cor-knot (LSI Solutions) is used to gently tie the outer thread.

Before the aortic clamp, the “U” stitch made with a pledgeted 4-0 polyvinylidene fluoride suture is placed for insertion of the ACP cannula. After completion of the mitral valve repair, aortic declamping and venting of the air in the left ventricle are performed. At our institution (Nagoya University Graduate School of Medicine, Nagoya, Japan), decannulation and hemostasis at the ACP cannulation site have been performed with robotic assistance. First, a tourniquet without tying is used to tighten the “U” stitch placed at the time of insertion. Next, the pursestring suture is placed with a pledgeted 2-0 polyester suture that surrounds the same site, with the needle running only to the adventitia (Figure 1). Third, to confirm that bleeding is under control, a tourniquet is used to tighten the outer thread after loosening the inner thread (Figure 2). Fourth, the Cor-knot is used to gently tie the outer thread (Figure 3). Finally, a knot-pusher is used by the patient’s side surgeon to tie the inner thread. Once hemostasis is confirmed, the patient is weaned from the heart-lung machine (Video).

## Comment

The effectiveness of the Cor-knot automated fastener has become apparent.^3^ In particular, it has become widely used in minimally invasive endoscopic cardiac surgery, and the same is true for robotic cardiac surgery. Notably, the attachment pressures of the Cor-knot are stronger than those of the knot pusher,^1^ a property that helps prevent paravalvular leakage and bleeding. In general, the Cor-knot is indicated for use for approximation of soft tissue and prosthetic materials, such as the annuloplasty ring and valve. However, its use has become increasingly varied. Furthermore, use of this automated fastener for distal anastomosis in open aortic arch surgery^4^ and for hemostasis at the ACP cannulation site in minimally invasive direct-vison mitral valve surgery^5^ has been reported. However, minimally invasive direct-vison mitral valve surgery does not necessary need an automated fastener. Hemostasis at the ACP cannulation site is more important in robotic mitral valve surgery because of limited visraion. At our institution, decannulation and hemostasis at the ACP cannulation site are performed with robotic assistance. Therefore, movement of the needle for the additional suture can be performed smoothly. However, suture tying must be performed with a knot pusher by the patient’s side surgeon, and same-site bleeding control is sometimes difficult. Therefore, we decided to use the Cor-knot automated fastener. Because of the fastener’s strong attachment pressures, the needle should be run only in the adventitia to avoid tearing of the aortic wall. The surgeon also needs to deploy the Cor-knot gently. We have performed this procedure in almost 30 cases and have not observed perforation of the aorta or formation of a pseudoaneurysm. Caution must be exercised with regard to aortic perforation given the strong attachment pressures of the Cor-knot fastener. To avoid these complications, the patient’s side surgeon must be proficient in handling the Cor-knot. We believe that it is very important to be aware of the pitfalls and to use good technique when using the Cor-knot. In summary, we propose a reasonable technique that uses the Cor-knot automated fastener. This technique has allowed us to obtain speedy and reliable hemostasis at the ACP cannulation site in robotic cardiac surgery.
